# Case report: Advanced breast cancer with scalp metastases: a report of two cases

**DOI:** 10.3389/fonc.2024.1382415

**Published:** 2024-10-21

**Authors:** Jiaxuan Yu, Tianze Yao, Min Zhang, Bingxin Li, Yongqiang Yao

**Affiliations:** ^1^ Health Management Center, Affiliated Zhongshan Hospital of Dalian University, Dalian, China; ^2^ Department of Clinical Medicine, Jinzhou Medical University, Jinzhou, China; ^3^ Department of Breast and Thyroid Surgery, Affiliated Zhongshan Hospital of Dalian University, Dalian, China

**Keywords:** breast cancer, end-stage breast cancer, metastasis, metastatic breast cancer, cutaneous metastasis

## Abstract

**Background:**

Breast cancer, identified as the most prevalent cancer worldwide, presents considerable difficulties in advanced stages, especially when involving metastatic spread. Scalp metastasis from breast cancer represents a rare and insufficiently explored occurrence. This paper seeks to illuminate this uncommon manifestation by presenting two cases of scalp metastatic breast cancer in Chinese women.

**Case report:**

Case 1: A 45-year-old Chinese woman with a history of invasive ductal carcinoma presented with a scalp lesion indicative of recurrence. Concurrently, she was diagnosed with bone metastases and recurrence at the original site. Despite undergoing various treatments, including chemotherapy and hormonal therapy, her condition worsened, ultimately leading to her passing. Case 2: A 40-year-old Chinese woman was initially diagnosed with bilateral breast invasive mucinous carcinoma presenting with bilateral breast masses and a scalp lesion. She also had multiple bone metastases. Following chemotherapy and hormonal therapy, her disease stabilized.

**Conclusion:**

These cases of scalp metastatic breast cancer underscore the complexities involved in managing advanced stages of the disease, especially with rare metastatic manifestations. They highlight the importance of comprehensive diagnostic methods, encompassing full-body skin evaluations, and draw attention to the socioeconomic challenges faced in cancer treatment. These findings point to the necessity for more targeted research on uncommon metastatic forms in breast cancer aiming to enhance patient outcomes and refine management approaches.

## Introduction

Breast cancer is a significant concern in women’s health, with a considerable impact on individuals and healthcare systems globally. The World Health Organization (WHO) has reported that it is the world’s most prevalent cancer given that there were 7.8 million women alive with a breast cancer diagnosis as of the end of 2020 (1). Though the death rates from breast cancer have been steadily decreasing since 1989, it is still the second leading cause of cancer death in women (2). Based on the Surveillance, Epidemiology, and End Results (SEER) database, the overall 5-year relative survival rate in patients with localized and regional diseases was 99% and 86%, respectively. However, those with distant metastases had a lower rate of 30% (3). Thus, addressing the unmet need for patients with distal metastatic diseases remains a significant topic.

The most common distal locations for metastatic breast cancer are the bones, lungs, brain, and liver (3–5). Cutaneous metastases from internal carcinoma are rare; reportedly, approximately 23.9% of them originate from breast cancer (6, 7). The exact prevalence of skin metastases among breast cancer patients remains unknown, but it is frequently associated with poorer outcomes (8). Scalp metastatic breast cancer is an even rarer presentation of skin involvement; there have only been limited case reports (9–11), and its clinical characteristics and prognostic value have not been thoroughly investigated. Here, we aimed to contribute to adding evidence to this subset of patients by reporting two cases of scalp metastatic breast cancer.

## Case report

### Case 1

A 45-year-old Chinese female presented with an enlarging left parietal skin lesion in December 2019. She denied any other symptoms and notable masses. She had no pertinent medical, surgical, or family history. She also did not have any specific breast cancer risk factors. Before the onset of scalp lesions, she had a complicated medical history of left recurrent invasive ductal carcinoma (IDC). It was initially diagnosed in 2016 with a size of 1.3 × 1.3 × 1.3 cm. Pathology showed Ki-67 of 20%, positive estrogen receptors (ER), progesterone receptors (PR), and negative ErbB2 receptors (formerly known as HER-2). She underwent left lumpectomy with axillary lymph node dissection, followed by adjuvant chemotherapy (docetaxel, epirubicin, and cyclophosphamide) and localized radiation therapy. She had been on toremifene maintenance. In 2018, a 2.0 × 1.0 × 1.5-cm focus was noticed underneath the previous surgical scar, and it was confirmed to be recurrent IDC by core needle biopsy. The bone scan revealed possible metastases in multiple areas, and whole-body magnetic resonance imaging (MRI) did not find other suspicious lesions. She then underwent bilateral oophorectomies for ovarian suppression and switched to maintenance anastrozole and zoledronic acid.

**Figure 1:**
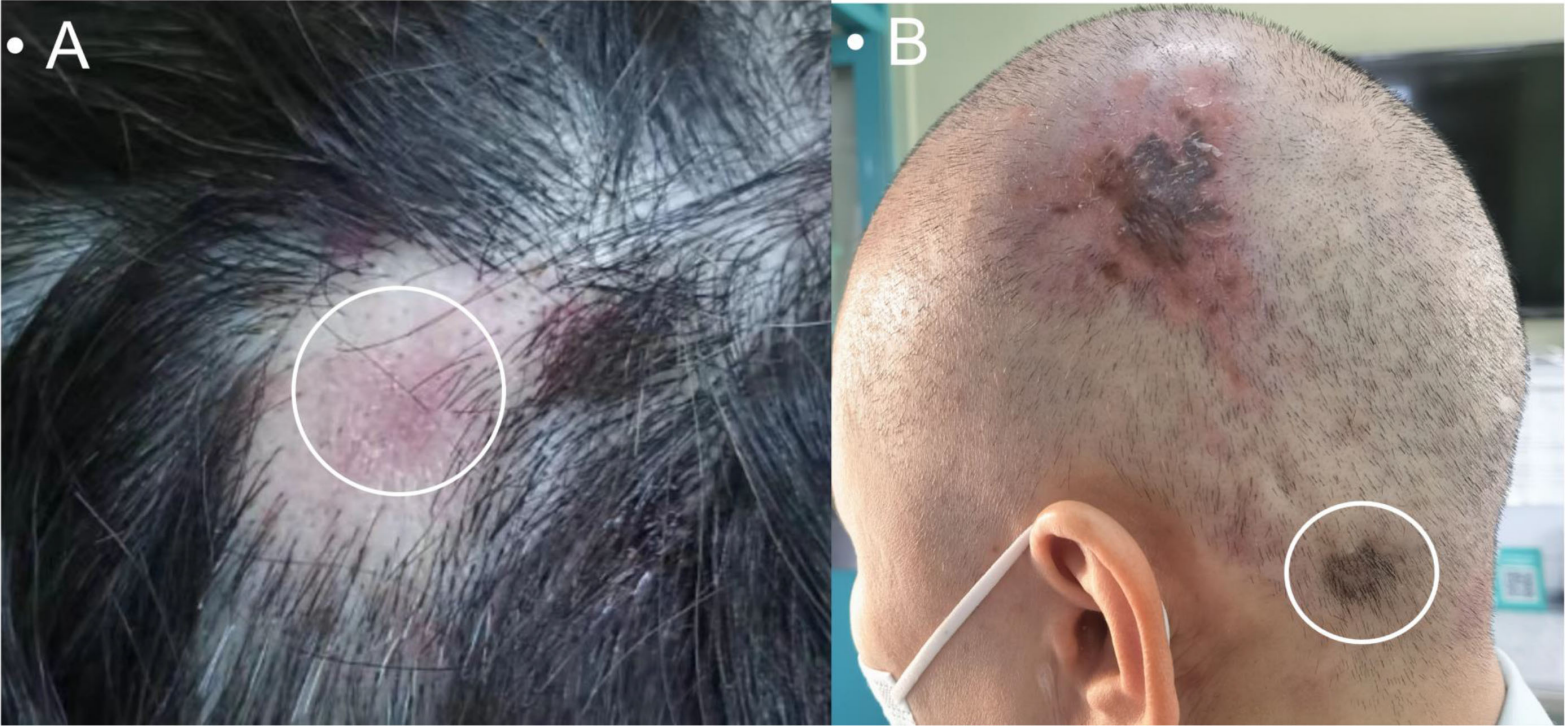
A 2.5 × 2.0 × 0.5-cm flat hard, well-demarcated erythematous lesion without ulcerations on the top of the scalp **(A)**; a new lesion on the left occipital region **(B)**.

**Figure 2:**
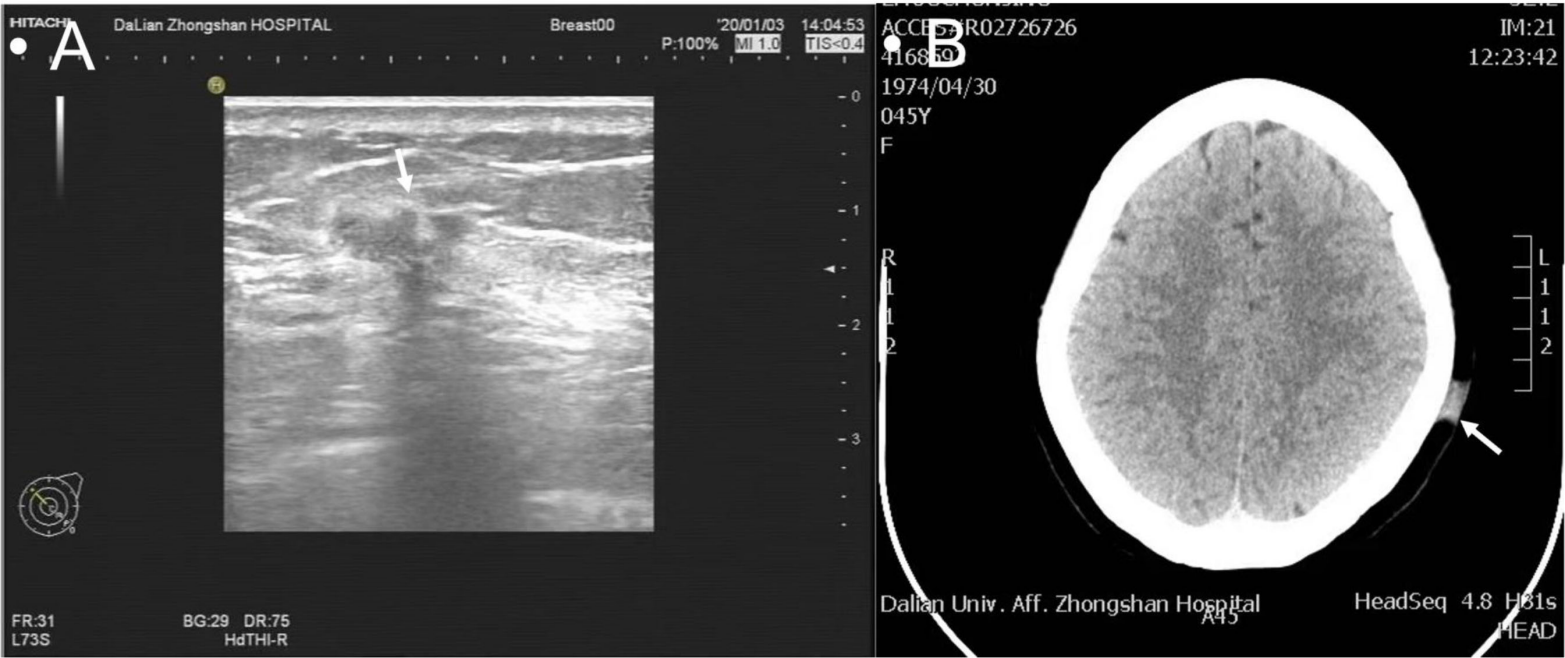
Multiple nodular masses with calcifications on ultrasound classified as BI-RADS 6 **(A)**; a 1.3-cm hyperdense patchy lesion on CT scan **(B)**.

Upon the recent scalp presentation, the physical exam showed a 2.5 × 2.0 × 0.5-cm flat hard, well-demarcated erythematous lesion without ulcerations (**Figure 1A**). Left breast ultrasound revealed multiple nodular masses with calcifications [breast imaging report and data system (BI-RADS) category 6], and head computed tomography (CT) showed a 1.3-cm hyperdense patchy lesion (**Figures 2A, B**). Dermatology was consulted, and a biopsy was obtained. The final pathology reported skin metastatic breast cancer with Ki-67 of 20%, positive ER, PR, and negative ErbB2 (**Supplementary Figures 1A–D**). The patient was eventually diagnosed with Stage IV (rpT1cN1M1) recurrent IDC.

The patient and family initially refused further chemotherapy as well as CDK 4/6 inhibitors, exemestane, and everolimus. She was switched to single-agent fulvestrant and continued zoledronic acid. In November 2020, she had a noticeable enlargement of the previous scalp lesion and a new one on the left occipital region (**Figure 1B**) accompanied by intermittent pain. In addition to worsening bony involvement and left breast recurrent foci, multiple lymph nodes in the right axillary and left supraclavicular region were seen. The new scalp lesion was biopsied, and the pathology showed metastatic breast cancer with Ki-67 of 40%, positive ER, PR, and low ErbB2. After completing eight cycles of salvage chemotherapy (paclitaxel and capecitabine), the skin lesion improved; however, the metastatic bone diseases, left recurrent foci, and lymph nodes did not show any response. We recommended multidisciplinary team (MDT) consults for further management, but she and her family refused due to financial reasons. In early 2022, the patient passed away due to multiorgan failure and cachexia.

### Case 2

**Figure 3:**
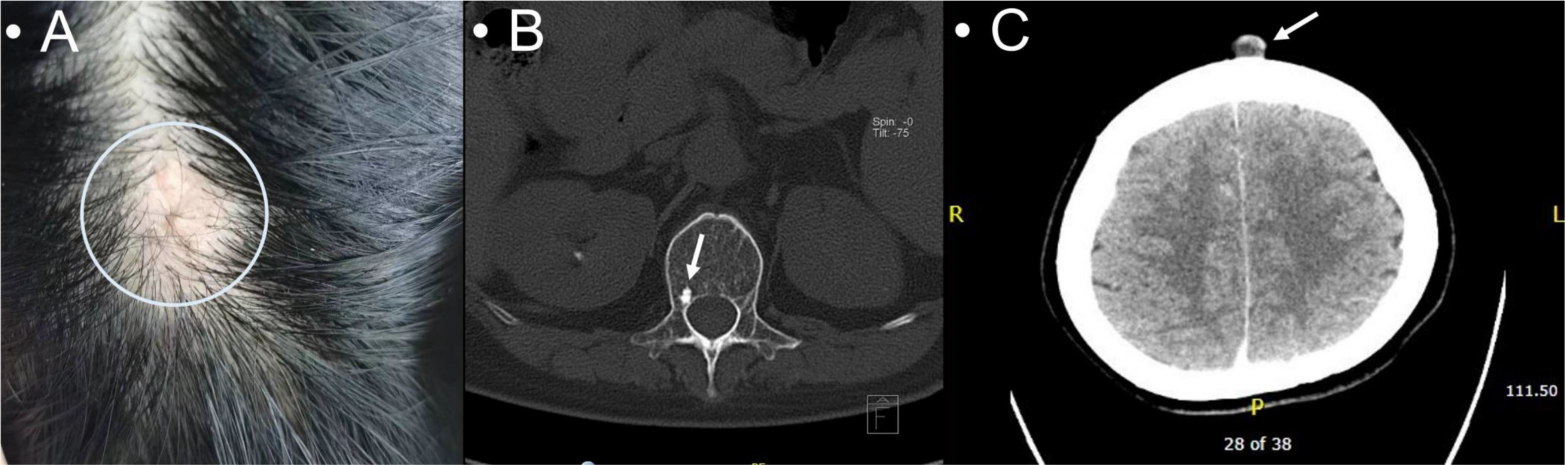
A 2.0 × 0.5-cm non-mobile non-erythematous elevated poorly demarcated nodule in the occipital scalp **(A)**; hyperdensity in the 1st lumbar spine suspicious for metastases **(B)**; a cutaneous occipital scalp nodule on the CT scan **(C)**.

This case involved a 40-year-old Chinese woman with bilateral breast masses and occipital scalp lesions. In July 2020, she underwent evaluation for palpable masses in both breasts presenting with no nipple discharge or skin changes. She reported no other symptoms. Her past surgical history included a partial hip replacement and a left salpingo-oophorectomy. She did not have any pertinent medical or family history. She had no specific risk factors related to breast cancer. Physical examination revealed several lumps: a 2.0 × 2.0-cm firm, non-mobile mass with poorly defined borders in the right breast’s outer lower quadrant; a 3.5 × 2.5-cm mass with similar characteristics in the left upper medial region; a 2.5 × 1.5-cm enlarged node in the right axillary region; no significant enlarged lymph nodes on the left; and a 2.0 × 0.5-cm non-mobile nodule in the occipital scalp region (**Figure 3A**). A diagnostic mammogram showed multiple nodules with calcifications in both breasts (BI-RADS 4a-b). Breast ultrasound identified several hyper- and hypoechoic masses with calcifications, the largest being 2.7 × 2.5 × 2.7 cm in the right breast (BI-RADS 4c) and 3.7 × 2.0 × 3.9 cm in the left breast (BI-RADS 4b). A full-body CT scan revealed enlarged right axillary lymph nodes, compression fractures in the 12th thoracic spine, and a hyper-dense area in the 1st lumbar spine, suspicious for metastases. She underwent an ultrasound-guided vacuum-assisted excision of the right breast mass. She also had right axillary lymph nodes and occipital scalp nodules (**Figures 3B, C**). The pathology reports were consistent across all samples indicating invasive mucinous carcinoma with positive hormonal receptors (ER and PR), low Ki-67, and negative ErbB2 (**Supplementary Figures 2A–D**). She was ultimately diagnosed with right Stage IV (pT3N1M1) breast mucinous carcinoma and left Stage IV (pT2N0M1) breast mucinous carcinoma.

Systemic therapy was started promptly, and the patient received six cycles of docetaxel, epirubicin, and cyclophosphamide. She was then on goserelin for ovarian suppression and zoledronic acid for metastatic bone diseases. In December 2020, the patient underwent a right salpingo-oophorectomy and had been on maintenance anastrozole. After completing the chemotherapy, ultrasound revealed slightly decreased sizes of breast masses, while all other follow-up imagines remained stable. We recommended CDK 4/6 inhibitors with aromatase inhibitors or single-agent fulvestrant, but the patient and her family refused. She remained on anastrozole and zoledronic acid and followed up regularly at a local hospital. She contacted in December 2023 and reported a stable condition.

**Figure 4:**
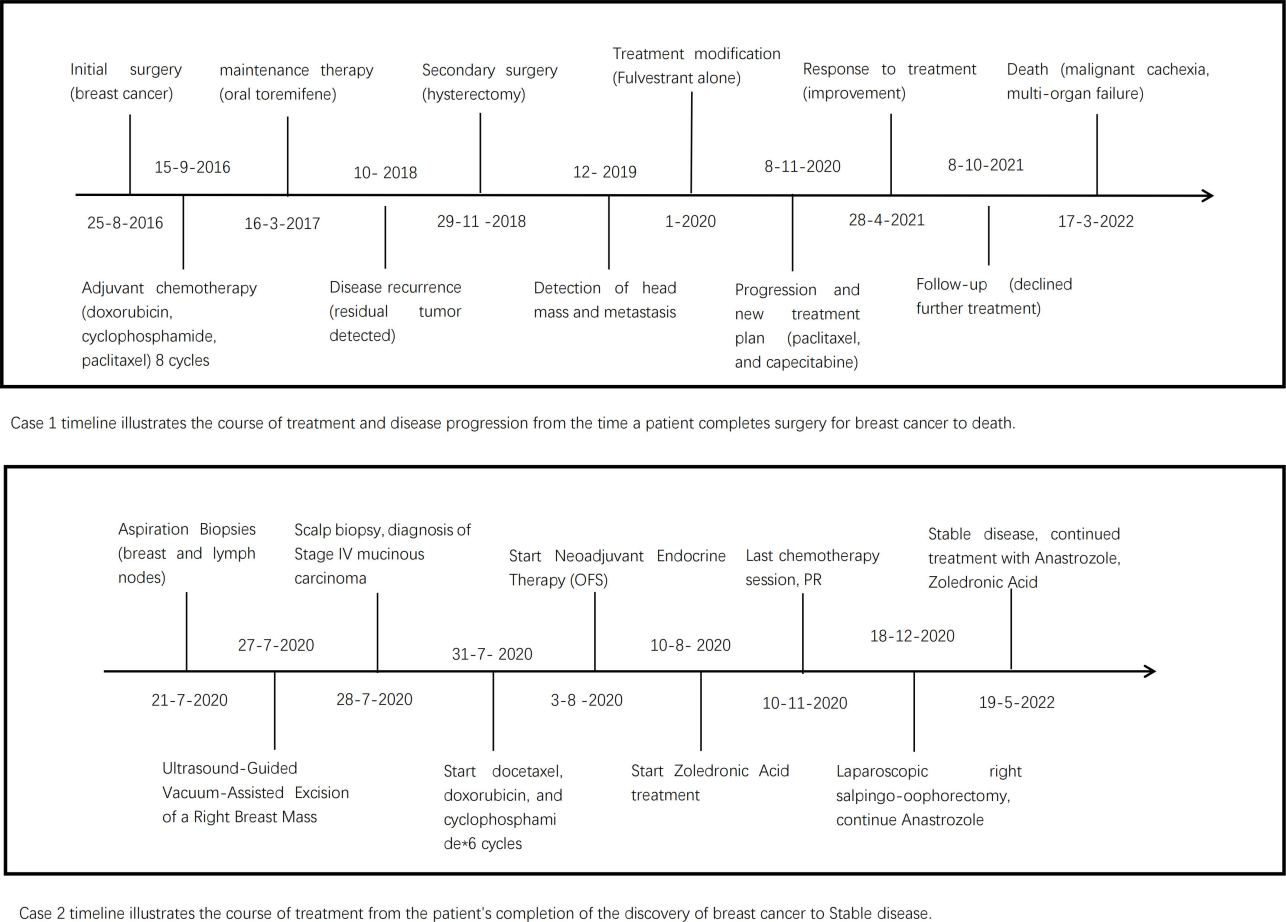
Summary of timelines of both patients.

We have included a detailed timeline of relevant events, including surgery time and a chemoradiation treatment course (**Figure 4**).

## Discussion

In both cases, the patients had aggressive hormonal receptor-positive (ER and PR) ErbB2-negative breast cancers with nodal involvement and distant metastases. One patient presented with invasive ductal carcinoma, while the other had mucinous carcinoma. Physical examinations revealed palpable nodules in all scalp lesions. Differential diagnoses encompass inflammatory diseases, lipoma, benign cutaneous tumors, primary cutaneous malignancies, and metastatic diseases from other origins. Skin biopsies were performed on both patients underscoring the significance of comprehensive skin exams in advanced-stage breast cancer cases. Notably, both patients exhibited symptomatic bone metastases and skin involvement suggesting a poorer prognosis. However, further data and studies are necessary for definitive conclusions. Financial constraints led both patients to decline positron emission tomography/computed tomography (PET/CT) for extended cancer assessment and recommended treatments highlighting the socioeconomic barriers facing patients with advanced cancers in developing countries. The outcomes differed between the two patients, with one deceased and the other with stable disease. The reasons for these divergent outcomes could be multifactorial, potentially linked to the cancer subtype.

Scalp metastases originating from non-cutaneous primary malignancies, such as lung, ovarian, gastric, or renal cell carcinoma (12–15), are a relatively rare phenomenon. There is limited comprehensive data available regarding its exact prevalence and prognosis. While the exact prevalence remains unclear due to the scarcity of comprehensive studies, it is generally considered uncommon. For instance, a study by Brownstein and Helwig reported that scalp metastases account for approximately 4% of all cutaneous metastases (16). The clinical characteristics and prognosis of scalp metastases in these cases can vary widely depending on the primary cancer type, stage at the time of diagnosis, and individual patient factors. Gupta’s team reported scalp lesions as an initial presentation of an aggressive lung adenocarcinoma in a patient who had no response to chemoradiation therapy (17). Ryu and his team reported that a scalp metastasis case originated from a primary gastric adenocarcinoma (3). After upgrading the tumor stage and systemic therapy, the patient underwent palliative surgery and unfortunately passed away a year after diagnosis. Though no extensive cohort studies have investigated this, scalp involvement appears more frequently in advanced stages and recurrent diseases indicating the linkage to a poorer outcome.

Like other primary origins, scalp metastasis from invasive breast cancer is also a rare presentation; there have been only case reports of this complication (9–11, 18, 19). Costa’s team reported a scalp metastatic case in a 66-year-old female; however, imaging modalities, including diagnostic mammogram and PET/CT, did not show any pertinent breast axillary abnormalities (19). Alizadeh et al. also reported a similar case in a 44-year-old female with metastatic breast carcinoma. However, no concrete disease foci were identified on MRI, CT, or ultrasound (10). Besides the presentation in possible occult breast cancer cases, Ciazynska et al. reported a case of occipital scalp lesion in a 47-year-old female, with the scalp nodule being the initial presentation of left breast cancer confirmed by CT and pathology (20). Both of our cases had definite evidence of primary breast foci, and the major difference compared to others was that the scalp findings were signs of recurrence and concurrent findings instead of the initial presentation. Abdelhafeez et al. also reported scalp lesions as a sign of recurrence of breast cancer (21). These all highlight that scalp metastatic breast cancer has various presentations along the disease course. Thus, a full-body skin examination by a dermatologist is crucial when evaluating a suspicious new skin lesion in advanced breast cancer patients. Performing a dermoscopy exam and skin biopsy can help establish a definitive diagnosis, and sometimes, it can assist with tumor staging. Given limited data, no standard treatment options have been proposed. However, in the case of metastatic disease, systemic treatment might be a more optimal choice. The value of local surgical and topical treatment remains unknown.

Prognosis and outcomes for patients with scalp metastases from breast cancer are often contingent upon disease extent, treatment response, and the presence of concurrent metastases in other organs. A case similar to ours, reported by Abdulraheem and colleagues, described a patient with recurrent ER-positive breast cancer and multiple distant metastases (11). Despite undergoing extensive chemoradiation therapy, the patient experienced poor outcomes. Conversely, the case published by Abdelhafeez’s team showed 1 year of disease-free survival following chemoradiation therapy and secondary hormonal treatment. There is a clear need for further research and more extensive studies to gain a more precise understanding of the prevalence and prognosis of scalp metastatic cancer in breast cancer patients. This would enable more informed clinical decision making and enhance management strategies.

## Conclusion

The cases presented here, involving scalp metastatic breast cancer in two Chinese women, underscore a rare yet significant facet of breast cancer progression. These instances, marked by late-stage, hormone receptor-positive, and ErbB2-negative cancers, highlight the intricacies and challenges associated with treating advanced breast cancer that manifests with unusual metastatic presentations. Furthermore, these cases reflect the profound influence of socioeconomic factors on treatment choices and outcomes shedding light on the frequently overlooked obstacles in managing advanced cancer cases. There is a pressing need for more inclusive and comprehensive research, mainly focused on understanding and managing the rare metastatic forms of breast cancer, to enhance prognosis and patient care.

## Data Availability

The original contributions presented in the study are included in the article/**Supplementary Material**. Further inquiries can be directed to the corresponding author.

## Supplementary material

Supplementary Figure 1 Scalp skin biopsy with hematoxylin and eosin staining showing invasive ductal carcinoma **(A)**; estrogen receptor-positive with moderate nuclear staining intensity **(B)**; progesterone receptor positive with strong nuclear staining intensity **(C)**; ErbB2 receptor negative **(D)** (×400).

Supplementary Figure 2 Scalp skin biopsy with hematoxylin and eosin staining showing invasive mucinous carcinoma **(A)**; estrogen receptor positive with strong nuclear staining intensity **(B)**; progesterone receptor positive with strong nuclear staining intensity **(C)**; ErbB2 receptor negative **(D)** (×100).
