# Supplementary figures and images for: Case report: Advanced breast cancer with scalp metastases: a report of two cases
Source: Front Oncol. 2024 Oct 21;14:1382415. doi: 10.3389/fonc.2024.1382415 (PMC11532104; doi:10.3389/fonc.2024.1382415)

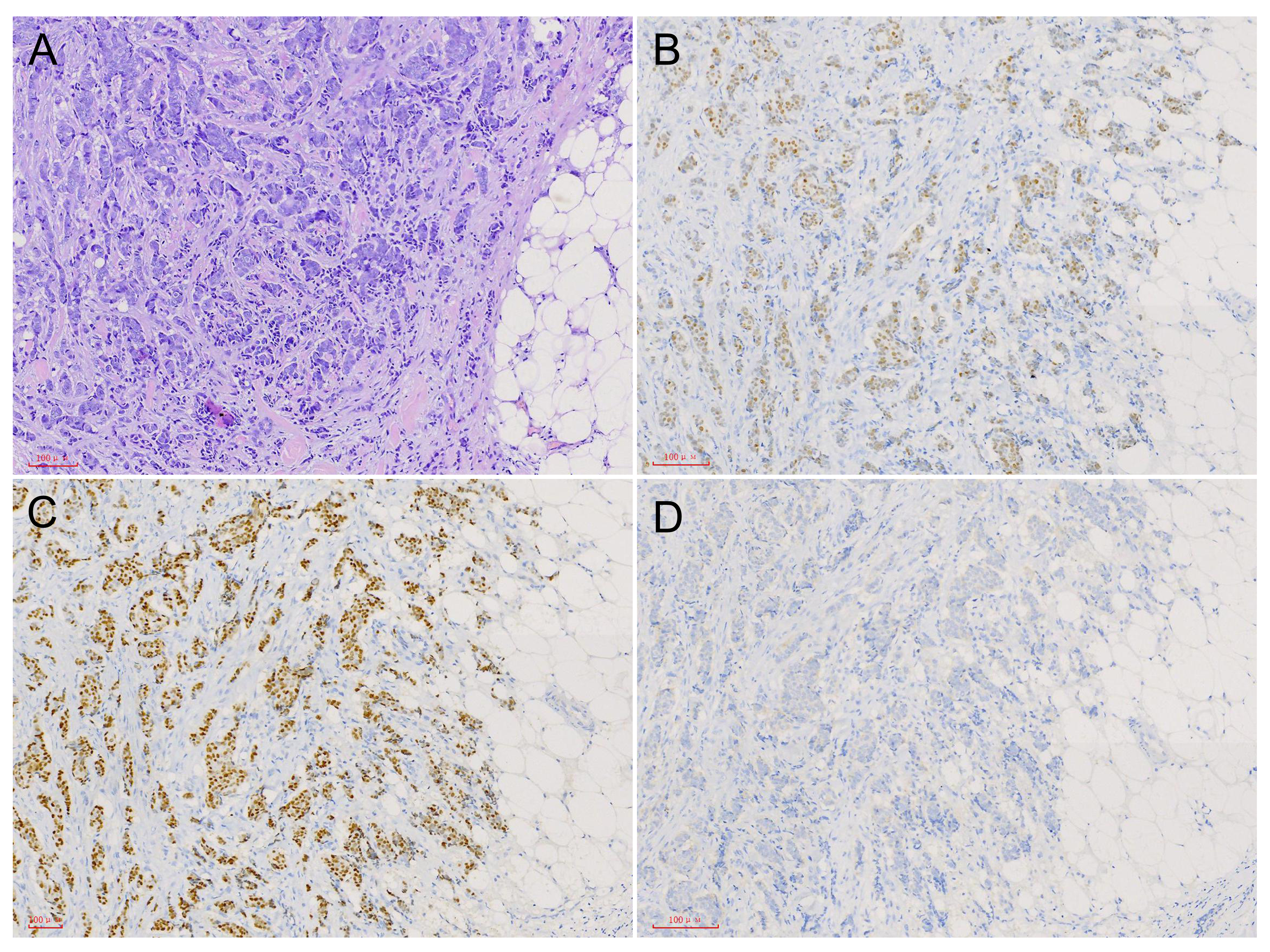

Supplement: Supplementary Figure 1 — Scalp skin biopsy with hematoxylin and eosin staining showing invasive ductal carcinoma (A); estrogen receptor-positive with moderate nuclear staining intensity (B); progesterone receptor positive with strong nuclear staining intensity (C); ErbB2 receptor negative (D) (×400). [file Image1.tif]

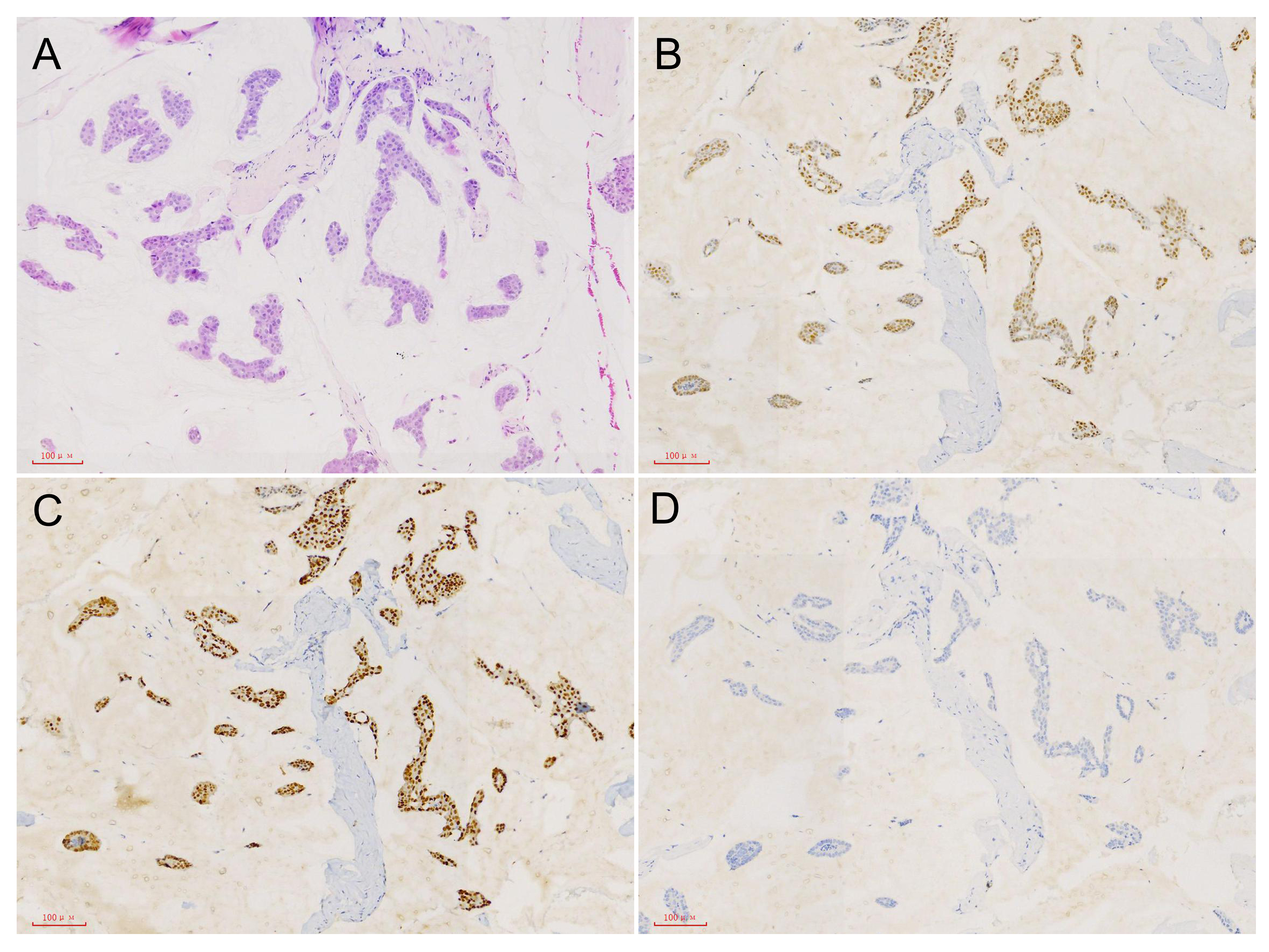

Supplement: Supplementary Figure 2 — Scalp skin biopsy with hematoxylin and eosin staining showing invasive mucinous carcinoma (A); estrogen receptor positive with strong nuclear staining intensity (B); progesterone receptor positive with strong nuclear staining intensity (C); ErbB2 receptor negative (D) (×100). [file Image2.tif]
